# Does functional planning, 3D templating and patient-specific instrumentation improve accuracy in total hip replacement?— a randomized controlled trial

**DOI:** 10.1186/s42836-022-00143-6

**Published:** 2022-10-02

**Authors:** Christopher Thomas, Vatsal Gupta, Helen Parsons, Andrew Metcalfe, Pedro Foguet, Richard King

**Affiliations:** 1 University Hospitals Coventry & Warwickshire NHS Trust, Coventry, England; 2 Present address: Dorset County Hospital, Williams Avenue, Dorchester, DT1 2JY UK; 3 University of Warwick, Coventry, England

**Keywords:** Total hip arthroplasty, Functional templating, Spine-pelvic, Navigation, Patient-specific instrumentation

## Abstract

**Aims:**

Debate continues as to the optimal orientation of the acetabular component in total hip arthroplasty (THA) and how to reliably achieve this. The primary objective of this study was to compare functional CT-based planning and patient-specific instruments with conventional THA using 2D templating.

**Methods:**

A pragmatic single-center, patient-assessor blinded, randomized control trial of patients undergoing THA was performed. 54 patients (aged 18–70) were recruited to either Corin Optimized Positioning System (OPS) or conventional THA. All patients received a cementless acetabular component. All patients underwent pre- and postoperative CT scans, and four functional X-rays. Patients in the OPS group had a 3D surgical plan and bespoke guides made. Patients in the conventional group had a surgical plan based on 2D templating X-rays. The primary outcome measure was the mean error in acetabular anteversion as determined by postoperative CT scan.

**Results:**

There was no statistically significant difference in the mean error in angle of acetabular anteversion when comparing OPS and conventional THA. In the OPS group, the achieved acetabular anteversion was within 10° of the planned anteversion in 96% of cases, compared with only 76% in the conventional group. The clinical outcomes were comparable between the groups.

**Conclusion:**

Large errors in acetabular orientation appear to be reduced when CT-based planning and patient-specific instruments are used compared to the standard technique but no significant differences were seen in the mean error.

## Background

Total hip arthroplasty (THA) is one of the most successful operations of all time [1], but for patients suffering complications, the consequences can be life changing. The success of THA has led to the procedure being offered to younger and more demanding patients, and the expectation of excellent long-term results are high. Mal-position of the components can result in impingement and edge loading, leading to complications such as dislocation and premature wear that can compromise survivorship [2–4].

The position of the acetabular component is one of the most demanding aspects of THA [5]. There are two related challenges when implanting the acetabular component: identifying the optimal component orientation and achieving it. A number of target values have been proposed [6–8]. More recently, the understanding of variations in acetabular anatomy and of the impact of spino-pelvic movement on *in-vivo* functional component orientation has rapidly evolved [9–12]. With it, the notion that a one-size-fits-all 'safe zone' for component orientation seems increasingly unlikely.

The second challenge is how to accurately reproduce the planned position. Beverland *et al*. reported on the reliability of the transverse acetabular ligament as a guide for acetabular component placement. However, even this relies on intraoperative judgement and freehand preparation [13]. Studies have shown significant inaccuracy when estimating the achieved component position intraoperatively [14]. Technology-driven systems have been developed to address these challenges; navigation and robotics have all been shown to improve accuracy in component position compared to conventional techniques, though they have mostly been used in the past to achieve a consistent cup position and not one that is patient-specific [15, 16].

The aim of this study was to compare the accuracy of acetabular component placement in THA performed with patient-specific instruments (PSIs) using functional 3D planning (the Corin Optimized Positioning System [OPS], Corin, UK) with THA performed with standard technique using 2D templating. Our secondary aims were to evaluate the clinical outcome of the OPS system compared to conventional THA using the same implants, to provide pilot data for a future large-scale randomized trial.

## Methods

### Study Design

We conducted a single-center, patient-assessor blinded, randomized controlled trial at a large university teaching hospital. Screening began on November 7, 2017. Randomization took place between January 5, 2018 and November 26, 2018. The trial was sponsored by the University Hospitals of Coventry and Warwickshire NHS Trust and funded by Corin Ltd, UK.

The study was formally registered on the International Standard Randomised Controlled Trial Number (ISRCTN) Registry with assignment number NCT03072706 on March 7, 2017 and was approved by the West Midlands (Solihull) NHS Research Ethics Committee on September 1, 2017.

### Eligibility and participant selection

Eligible patients were defined as adults aged 18–70 years, who could provide written informed consent and were due to undergo an elective primary total hip arthroplasty at the study site. Patients were excluded if they were deemed, by the treating clinician, unsuitable for a cementless acetabular component. Other reasons for exclusion included patients with significant orthopedic deformities, those receiving ionizing radiation treatment, pregnant women or women trying to become pregnant, patients enrolled into a clinical trial of an investigational medicinal product in the last 90 days, patients unable to undergo planning imaging required for the trial, and patients with complex anatomy where a planning CT was deemed necessary prior to surgery. To ensure that each procedure was independent, no bilateral cases were included.

Eligible participants were sent the patient information sheet via letter or e-mail. After a minimum of seven days to consider enrolment into the trial, patient understanding was checked and a suitably qualified professional from the research team obtained informed consent. Patient demographics and relevant past medical history were recorded, and patients completed three validated assessments: Hip Disability and Osteoarthritis Outcome Score (HOOS), Oxford Hip Score (OHS) and EQ-5D.

All patients underwent low-dose CT scan (mean dose of 2.8 to 4.0 mSv per scan). The CT protocol included the entire bony pelvis (top of iliac crest to 20 cm distal to the center of the femoral head; 1.25 mm slices), both knees (10 cm proximal to joint line to 10 cm distal to joint line; 2.5 mm slices), both ankles (5 cm proximal to distal tibia to 1 cm distal to bottom of foot; 2.5 mm slices), and scout (AP and lateral) images. All patients had four additional radiographs performed: one AP standing pelvic view and 3 lateral views incorporating the entire lumbar spine and pelvis, taken in 3 different positions; flexed seated, standing, and standing with 90° flexion of the contralateral hip [17]. This imaging was necessary for the planning, but was performed in all participants to maintain patient blinding.

Participants were then randomized in a 1:1 allocation to either THA, planned by the operating surgeon using 2D X-ray templating software, or to THA using CT planning, by Corin^TM^ OPS. Randomization was stratified by operating surgeon and in terms of patient BMI (BMI<30 *vs.* ≥30) and was performed by a computer algorithm at the Research and Development (R&D) Randomisation Unit at University Hospitals of Coventry and Warwickshire (UHCW) separate from the study team. Both the patients and the study team were blinded to treatment allocations, but it was not possible to blind the operating surgeon. To maintain blinding of the statistician performing the analysis, a randomization list was generated by a statistician independent of the project.

All patients underwent a repeat CT scan 6 weeks following surgery. Clinical assessment and outcome scoring were repeated 6 weeks, 4 months and 12 months after operation.

### Preoperative planning

#### Standard group

Templating was performed on preoperative supine AP pelvic radiographs employing 2D planning software (TraumaCad®, BrainLab) with the use of a radiographic scale marker to correct for magnification (KingMark®, BrainLab). The intended orientation of both acetabular and femoral components was recorded at the beginning of the operation. For this group, the acetabular target positions were 40 degrees of abduction and 20 degrees of anteversion as per Lewinnek *et al*. [8]. Femoral neck osteotomy heights were measured at 2D templating and cut according to this intraoperatively.

#### OPS group

Processing of imaging, preoperative plan selection and patient-specific instrumentation were performed according to the OPS method and performed by Corin in conjunction with the operating surgeon, as described by Pierrepoint *et al.* [18].

### Procedure

All procedures were performed by one of the senior authors (RK, PF) through a posterior approach. All patients received the same design of uncemented acetabular component (Trinity™, Corin). All patients in the trial underwent a postoperative CT scan to enable accurate assessment of component orientation and leg length.

#### Standard group

The components were positioned with a standard technique assisted by the use of mechanical alignment guides and referencing intraoperative landmarks, in particular, the transverse acetabular ligament. No reference was made to the preoperative CT scan.

#### OPS group

**Fig. 1:**
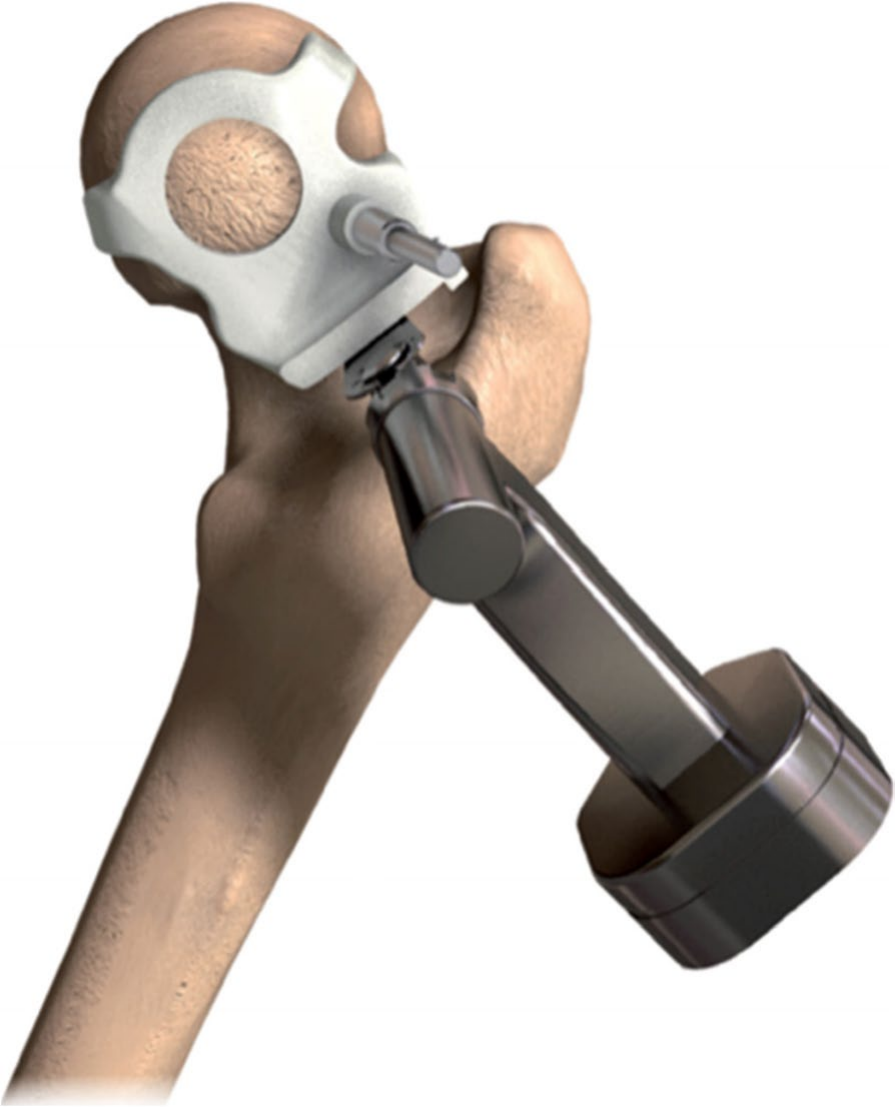
Femoral neck osteotomy cutting guide

**Fig. 2:**
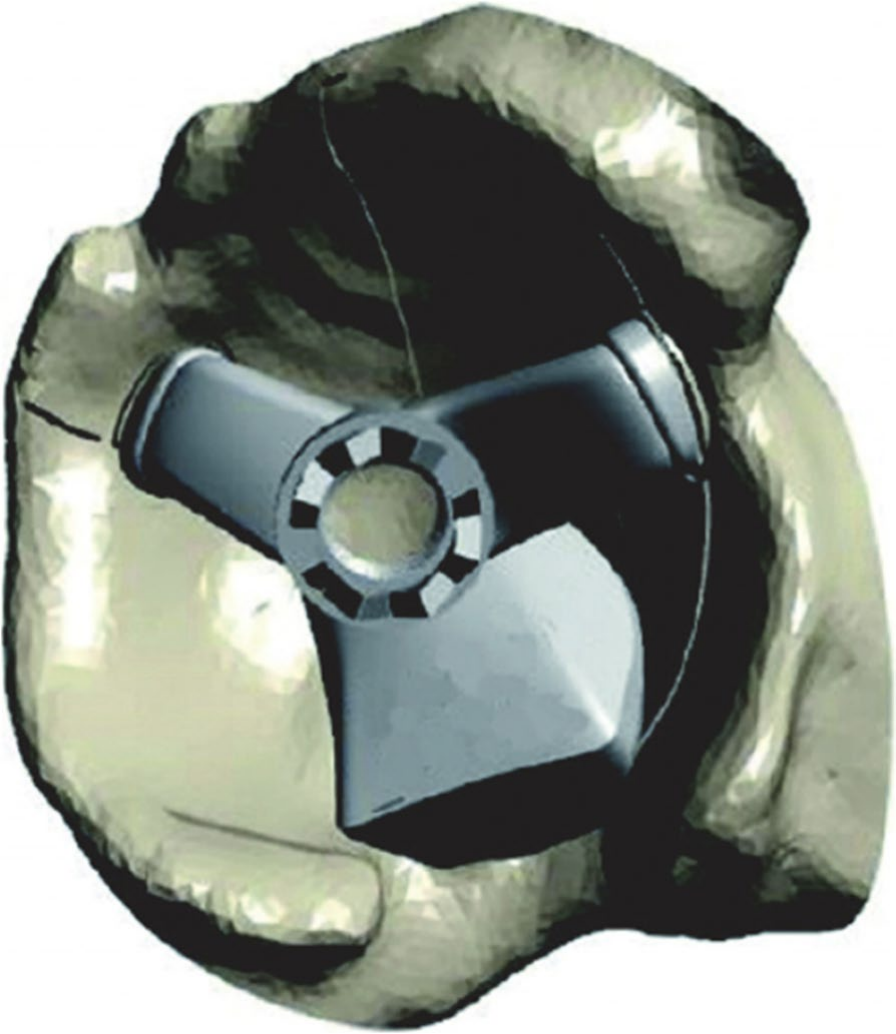
Acetabular orientation guide in provided 3D model

**Fig. 3:**
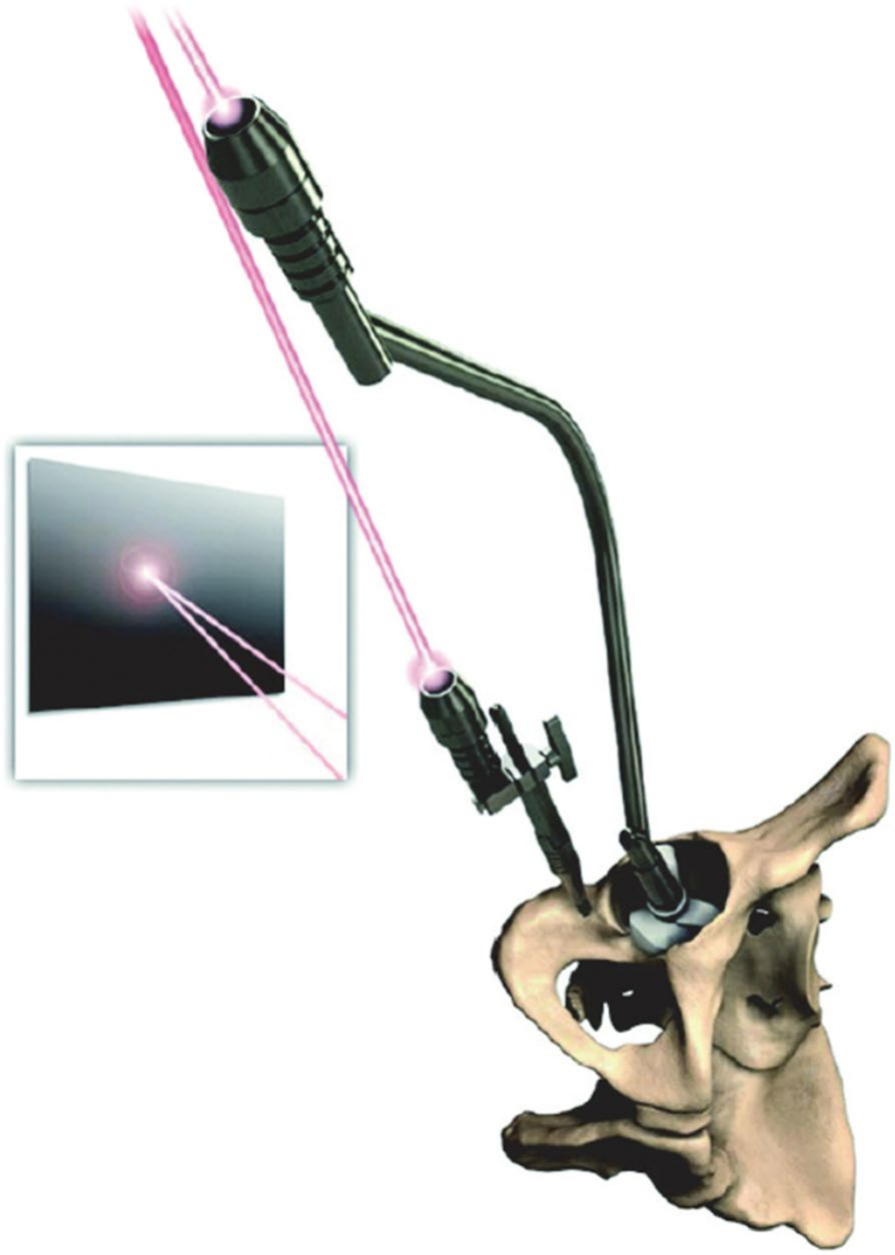
Fixed pelvic pin *in situ* with laser alignment with acetabular guide

**Fig. 4:**
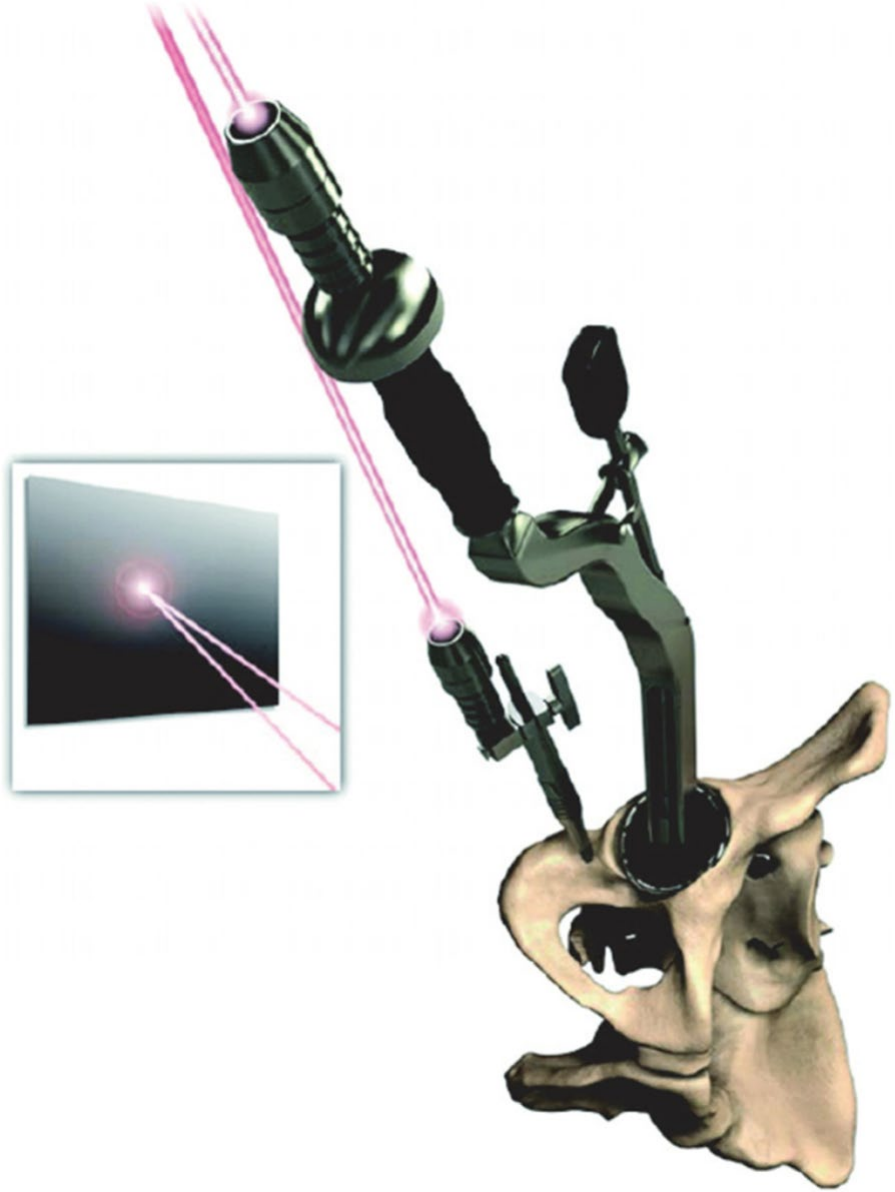
Acetabular component impactor with removable laser guide, matched to fixed pelvic target

After dislocating the hip, the femoral neck osteotomy was made using a standard oscillating saw-blade while the neck osteotomy PSI jig was held in place against the femur (Fig. 1). After acetabular exposure, the acetabular PSI was fitted carefully into the acetabulum after referencing the fit using the supplied acetabular model (Fig. 2). A laser pointer was then inserted into the acetabular PSI, thereby projecting a laser point onto the theatre ceiling (Fig. 3). A second “pelvic” laser pointer was fixed to the bony pelvis just next to the acetabulum, and orientated so that it matched the original acetabular laser point. The acetabular guide and laser were then removed while the pelvic laser was retained. The acetabulum was prepared with conventional reamers, with the pelvic laser indicating the axis of reaming. After trialing, the acetabular component was impacted after precisely aligning the cup inserter handle with the pelvic laser mark using an additional laser pointer that attached to the end of the acetabular inserter (Fig. 4).

### Outcome Measures

#### Primary Outcome Measure

The primary outcome measure was the mean error of planned and achieved acetabular cup anteversion, as assessed by postoperative CT scan. All measurements were made by a senior specialty trainee (CT) after extensive training and practice at Corin Group headquarters. The assessor was blinded to the treatment allocation. All postoperative scans in both treatment arms were analyzed twice, on separate occasions, and the mean of the results taken. All acetabular anteversion and abduction angles were measured with respect to the anterior pelvic plane, and then converted to the supine reference frame with respect to the preoperative CT scan using equations as described in Lembeck *et al*. [19]. The intra-observer reliability was calculated.

#### Secondary Outcome Measures

- The difference between planned and achieved acetabular cup abduction, assessed by postoperative CT scan;
- The difference between the surgeon's intraoperative estimated and achieved acetabular anteversion and abduction;
- The number of “outliers”, with an outlier defined as a difference of greater than 10° between the planned and achieved values for acetabular anteversion, abduction, or femoral version;
- Treatment efficacy as measured 6 weeks, 4 and 12 months after operation in terms of Hip Disability & Osteoarthritis Outcome Score (HOOS), Oxford Hip Score (OHS) and EQ-5D;
- Surgical procedure time as recorded in hospital electronic logs, encompassing positioning and preparation as well as 'skin-to-skin' time;
- Adverse events.

#### Sample Size and Statistical Analysis

The power was set at 90% and significance at 5%. Standard deviation of the difference in cup position was estimated at 10° based on several large studies reporting standard deviation in non-guided acetabular implant positioning for anteversion of 7–9 degrees [20, 21]. Minimal clinically important difference was estimated to be 10° based on Lewinnek's 'safe-zone' and non-OPS hips in this study having a target acetabular anteversion of 20 degrees [8]. Based on these assumptions, a sample size of 44 patients was needed. Allowing a projected 20% loss to follow-up, a final sample size of 54 patients was calculated to perform a two-sided test of superiority (27 in each allocation arm).

Descriptive statistics of means, ranges and standard deviations (SD) were calculated for continuous variables and count and proportions for categorical data. The mean of the primary outcome measure between treatment groups was assessed using *t*-tests and were considered to be statistically significant if the *P*-value was less than 0.05. Estimates of the treatment effects were calculated along with their 95% confidence intervals. Intra-observer reliability was calculated. No adjustment was made for multiplicity as acetabular anteversion angle was considered the primary outcome. All analyses were calculated on an intention-to-treat (ITT) basis. Statistical analyses were performed using R (v4.0.3, 2000, Vienna, Austria).

## Results

**Fig. 5:**
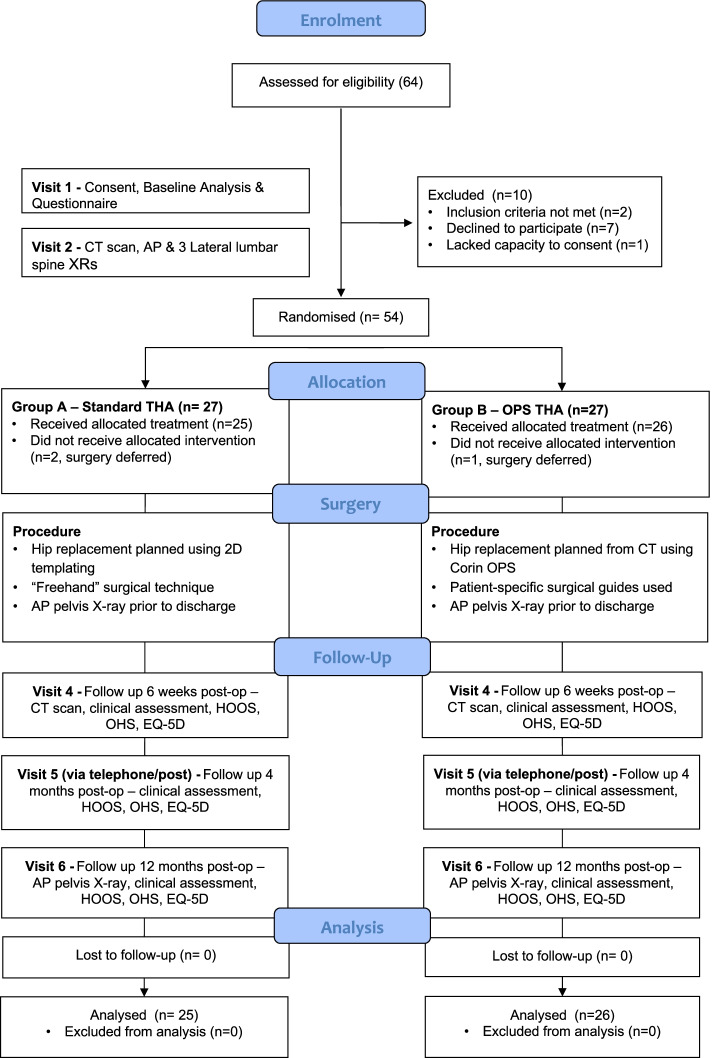
Consort diagram of participant flow throughout the study

A total of 64 patients were screened for eligibility and 10 were excluded as they did not meet the inclusion criteria or declined to participate. 54 participants were recruited and randomized into the study, with 27 allocated to each treatment arm (Fig. 5). The demographics of the two groups were balanced following randomization (Table 1). No patients withdrew from the trial after receiving their intervention. No serious adverse events were reported during the study. There were no revision procedures or significant harm events in either arm of the study. There were eight adverse events, seven (88%) of which were classed as unrelated to the study and one was classed as not assessable. There were no protocol violations.

**Table 1:** Patient descriptive statistics at baseline

	Group A Standard THA (***n***=27)	Group B OPS THA (***n***=27)	All (***n***=54)
Age at randomization (years, mean, SD)	57.3 (9.8)	58.8 (7.4)	58.1 (8.6)
Sex:Female (*n,* % of valid group)	12 (44.4)	18 (66.7)	30 (55.6)
Ethnicity: White British (*n*, %)	24 (88.9)	23 (85.2)	47 (87)
BMI (kg/m^2^, mean, SD)	29.7 (5.0)	30.3 (4.8)	30.0 (4.9)
Study Hip: Left (*n*, % of valid group)	9 (33.3)	10 (37.0)	19 (35.2)
Previous spinal surgery: Yes (*n*, % of valid group)	*	*	3 (5.7)
Known spinal deformity: Yes (*n*, % of valid group)	4 (15.4)	4 (14.8)	8 (15.1)
Neurological disorder: Yes (*n*, % of valid group)	2 (7.7)	2 (7.4)	4 (7.5)
Hip pathology: Osteoarthritis: Yes (*n*, % of valid group)	*	*	53 (98.1)
Length of legs: (*n*, % of valid group)			
About the same	14 (51.9)	15 (55.6)	29 (53.7)
Left leg longer	4 (14.8)	8 (29.6)	12 (22.2)
Right leg longer	4 (14.8)	2 (7.4)	6 (11.1)
Unsure	5 (18.5)	2 (7.4)	7 (13.0)
Walking aids: None (*n*, % of valid group)	17 (63.0)	19 (70.4)	36 (66.7)

### Primary Outcome

**Fig. 6:**
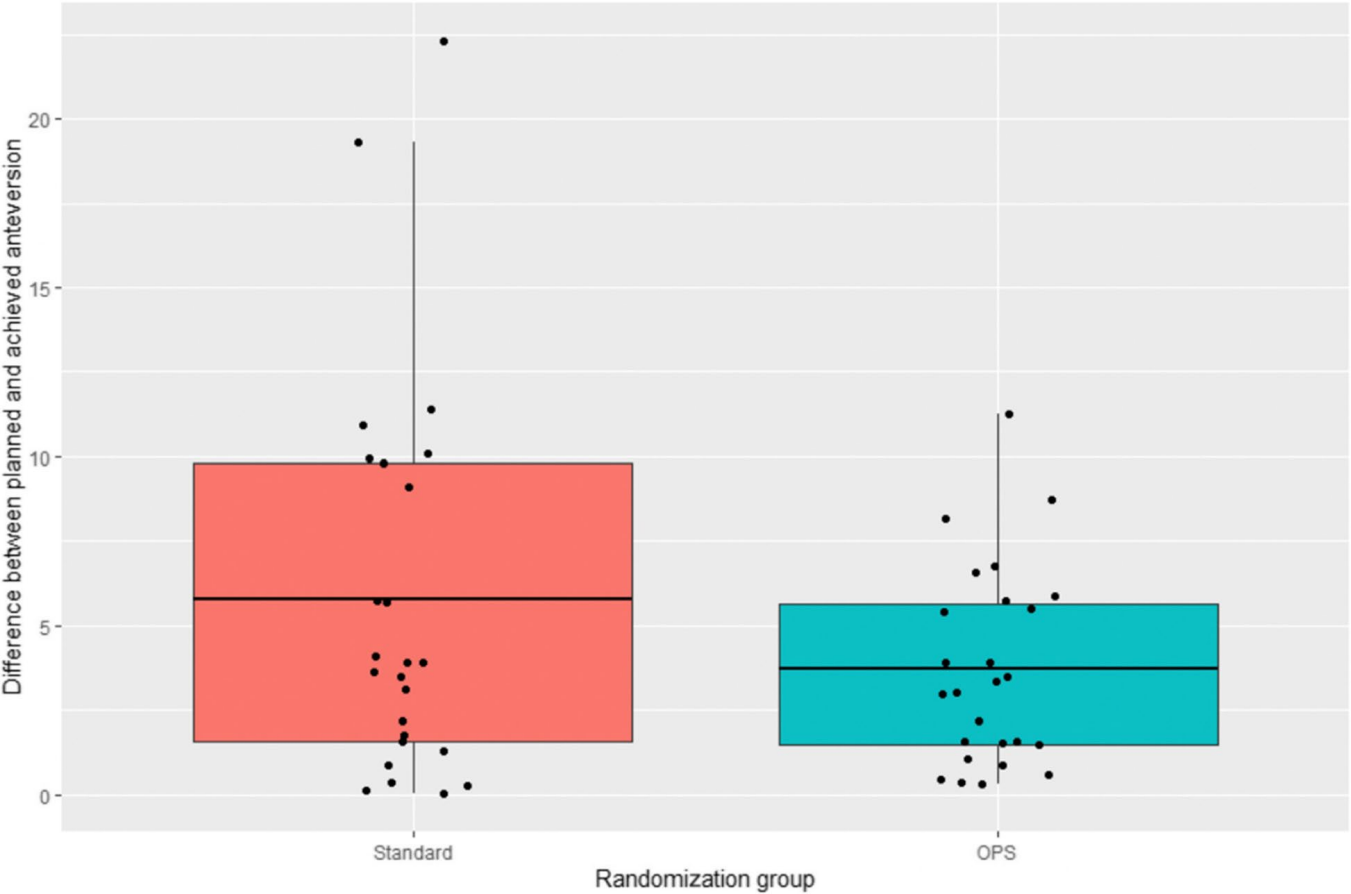
Box and Whisker plot of acetabular planned *vs*. achieved implant position in conventional and Corin OPS

The difference in mean error in angle of acetabular anteversion between the two groups was 2.08° (Standard *vs*. OPS; 95% CI: -0.56–4.73; *P*=0.1192) (Fig. 6).

### Secondary Outcomes

The difference in mean error in angle of acetabular abduction between the two groups was 1.07° (Standard *vs*. OPS; 95% CI: -1.19 – 3.33; *P*=0.3462). Planned and estimated *vs*. achieved implant positions are shown in Table 2. There were fewer outliers in the OPS group for acetabular anteversion, acetabular abduction and femoral version. Surgical time was increased by a mean of 8 minutes in the OPS group compared to the standard group. Questionnaire follow-up rates were 100% at baseline, 6 week and 12 month follow-up and 96% for the 4 month follow-up. There were no significant differences between the groups in any of the outcome scores at any stage (Table 3). There were no significant differences between the groups in postoperative leg length discrepancy or femoral neck osteotomy accuracy (Table 4).

**Table 2:** Difference between planned or (intraoperative) estimated and achieved component position with reference to 10 degree outlier cut-off

Implant position variable	Standard THA (***n***=25)	OPS THA (***n***=26)	Ratio (Std:OPS)
**Planning outliers**			
Difference between planned and achieved anteversion over 10° (*n*, % of valid group)	5 (20)	1 (4)	5
Difference between planned and achieved abduction over 10° (*n*, % of valid group)	4 (16)	2 (8)	2
Difference between planned and achieved femoral version over 10° (*n*, % of valid group)	8 (32)	3 (12)	2.7
Difference between planned and achieved acetabular anteversion OR abduction over 10° (*n*, % of valid group)	7 (28)	3 (12)	2.3
Difference between planned and achieved any angle of acetabular or femoral orientation over 10° (*n*, % of valid group)	11 (44)	5 (19)	2.3
**Estimation Outliers**			
Difference between estimated and achieved anteversion over 10° (*n*, % of valid group)	6 (24)	1 (4)	6
Difference between estimated and achieved abduction over 10° (*n*, % of valid group)	4 (16)	1 (4)	4
Difference between estimated and achieved femoral anteversion over 10° (*n*, % of valid group)	7 (26)	1 (4)	7
Difference between estimated and achieved acetabular anteversion OR abduction over 10° (*n*, % of valid group)	8 (32)	2 (8)	4
Difference between estimated and achieved any angle of acetabular or femoral orientation over 10° (*n*, % of valid group)	12 (48)	3 (12)	4

**Table 3:** Baseline, 6-week, 4- and 12-month follow-up HOOS, OHS and EQ-5D scores

Outcome	Time point	Standard (***n***=27)	OPS (***n***=27)	All (***n***=54)
**Hip disability and osteoarthritis outcome score (HOOS)**				
**HOOS: Pain**	Baseline	34.8 (14.1)	33.0 (14.0)	33.9 (13.9)
	6 weeks	79.8 (22.5)	83.7 (14.0)	81.8 (18.6)
	4 months	87.9 (18.0)	90.2 (12.0)	89.1 (15.1)
	12 Months	89.9 (18.4)	91.8 (15.3)	90.9 (16.8)
**HOOS: Symptoms**	Baseline	39.4 (18.9)	34.3 (15.1)	36.9 (17.1)
	6 weeks	77.4 (18.4)	80.8 (11.7)	79.1 (15.3)
	4 months	82.7 (16.9)	84.6 (11.9)	83.7 (14.5)
	12 Months	88.6 (16.4)	86.2 (16.8)	87.4 (16.5)
**HOOS: ADL**	Baseline	37.8 (19.0)	34.9 (18.1)	36.3 (18.4)
	6 weeks	74.1 (21.8)	79.5 (12.1)	76.8 (17.6)
	4 months	83.9 (18.1)	87.2 (14.2)	85.6 (16.1)
	12 Months	90.4 (18.8)	90.5 (17.7)	90.5 (18.1)
**HOOS: Sport/recreation**	Baseline	18.5 (20.8)	14.4 (15.9)	16.4 (18.5)
	6 weeks	47.5 (24.1)	58.4 (21.0)	53.1 (23.0)
	4 months	71.9 (27.1)	72.8 (24.7)	72.3 (25.6)
	12 Months	82.2 (25.2)	82.0 (24.5)	82.1 (24.6)
**HOOS: QoL**	Baseline	24.8 (16.9)	19.9 (15.1)	22.3 (16.1)
	6 weeks	55.2 (26.3)	57.7 (16.5)	56.5 (21.7)
	4 months	76.0 (21.8)	76.5 (24.5)	76.3 (23.0)
	12 Months	82.8 (20.7)	82.0 (20.0)	82.4 (20.2)
**Oxford Hip Score**				
**OHS**	Baseline	16.3 (6.3)	17.9 (7.9)	17.1 (7.1)
	6 weeks	30.9 (10.9)	35.7 (6.6)	33.4 (9.2)
	4 months	40.1 (9.3)	41.0 (7.3)	40.6 (8.3)
	12 Months	42.4 (9.8)	43.1 (7.3)	42.8 (8.5)
**EQ-5D**				
**EQ-5D**	Baseline	0.428 (0.235)	0.420 (0.261)	0.424 (0.246)
	6 weeks	0.691 (0.243)	0.748 (0.148)	0.720 (0.200)
	4 months	0.802 (0.201)	0.744 (0.289)	0.772 (0.249)
	12 Months	0.823 (0.180)	0.753 (0.232)	0.787 (0.209)

**Table 4:** Further secondary outcome measures

		Standard THA (***n***=27)	OPS THA (***n***=27)	All (***n***=54)
Absolute postop leg length difference (mm, mean, SD)	Hip	2.2 (1.8)	3.0 (2.5)	2.6 (2.2)
	Global	5.4 (3.8)	5.4 (5.4)	5.4 (4.6)
Absolute difference in osteotomy height (mm, mean, SD)	Planned and actual	2.9 (2.0)	1.9 (1.5)	2.4 (1.8)
	Estimated and actual	2.2 (1.9)	1.7 (1.3)	2.0 (1.6)
Surgery length (mean, SD)		1 h 29 m (12 m 44 secs)	1 h 37 m (21 m 3secs)	1 h 33 m (17 m 49 secs)

### Intra-observer reliability

The intraclass correlation coefficient (ICC) for anteversion was 0.99 (95% CI 0.983–0.995) and for acetabular abduction was 0.99 (95% CI 0.978–0.993). This suggests that the method of analyzing definitive acetabular position is extremely reproducible.

## Discussion

We found that there was no statistically significant difference between groups in our primary outcome, *i.e*., the error of acetabular anteversion. This might be because the study was insufficiently powered to detect the difference between groups. However, to our knowledge, there is no guidance on a clinically meaningful error of acetabular anteversion outside of Lewinnek's safe zone [20]. Furthermore, both the contributing surgeons to the study are experienced, high-volume primary and revision arthroplasty surgeons with a particular interest in acetabular component position. They were also experienced in using the Corin OPS prior to the commencement of the study. This may have resulted in the acetabular accuracy of the standard technique being superior to that more broadly achieved across clinical practice. It might be that if this study were repeated on a larger scale, incorporating lower volume and less experienced surgeons, the observed error would be larger and easier to detect. Also, this study did not capture long-term participant outcomes, which did not enable us to assess any possible effects that the use of OPS may have upon reductions in revision rates through improved stability and reduced edge loading. Doing so would require a much larger study, with much longer follow-up and, as such, is more likely to be borne out in registry data. Finally, whilst we have not shown a difference in this population, it does not preclude such an effect being present in subgroups, such as in patients at increased risk of instability [22].

We defined an outlier as a difference between planned and achieved component position > 10 degrees *post hoc.* This was in line with our target difference used to power the study. In an original paper by Lewinnek *et al*., acceptable acetabular position was defined as 20 degrees of anteversion and 40 degrees of abduction +/- 10 degrees [8]. This formed the basis of the target for the 2D templated cohort and guided the choice of the +/- 10 degrees outlier definition. There were more outliers in the standard care group than the OPS group for acetabular anteversion (20% *vs*. 4%), abduction (16% *vs*. 8%) and femoral anteversion (32% *vs*. 12%). Overall, 28% of patients in the standard treatment arm had an acetabular component that was > 10 degrees from the planned anteversion or abduction (12% for OPS). This is consistent with existing literature demonstrating similar frequencies of outliers in acetabular position [23]. It is also better than some reported literature on standard THA accuracy [24, 25]. The combined acetabular anteversion/abduction accuracy in the OPS treatment arm of this study was similar to a previously published series using OPS (89% *vs*. 91% respectively) [17]. Comparing estimated acetabular component position with the achieved position, the accuracy in abduction and anteversion increased in this study to 92%. This accuracy is also comparable with intraoperative image-guided and robotic total hip replacement studies [26, 27]. Possible explanations for the persistent 8% rate of outlying acetabular component positions include movement of the laser pointers due to osteoporotic bone or retractor tension or accepting 'imperfect' definitive component position to maximize primary press-fit. There was a mean increase in operating time of 8 minutes between the standard and the OPS treatment arm. This difference in mean operating time was not statistically significant and can be confounded by numerous other variables [28]. No significant differences were identified in any of our secondary patient-reported outcome measures. However, this was a pilot study with regard to secondary outcome measures.

The unique features of OPS are that it combines functional planning, 3D templating and patient-specific guides without the need for significant advanced financial outlay on additional equipment or staff training. Functional planning in the form of positional X-rays provides information regarding the change in pelvic tilt, accounting for the effect of spino-pelvic movements. This, in combination with the CT scan, allows for calculation of the primary arc of movement of the hip between sitting and standing for multiple combinations of acetabular anteversion, abduction, and femoral anteversion. The surgeon selects a bespoke acetabular orientation to minimize edge loading and impingement, thereby reducing the risk of instability and early wear. The 3D CT scan allows for manufacture of a custom-made targeting guide to ensure reproducibility of the planned position. Even if we could not demonstrate that the accuracy of OPS-guided total hip replacement is superior than with standard mechanical alignment guides, OPS total hip replacement is delivering a custom orientation based on functional templating and it may be that this results in reduced revision rates through reductions in edge loading and instability.

In conclusion, functional planning, 3D templating and patient-specific instrumentation using Corin OPS is a safe and reliable method of accurately placing the acetabular component in THA. Further research is needed to ascertain if functional planning impacts on patient outcomes, including long-term pain and function.

## Data Availability

The datasets used and/or analyzed during the current study are available from the corresponding author on reasonable request.
